# Contraceptive care for transgender and gender diverse individuals from the perspective of healthcare providers in Germany: a qualitative study

**DOI:** 10.1186/s12978-025-02223-7

**Published:** 2025-12-06

**Authors:** Charlotte Barton, Marie Werner, Lena Herrmann, Nadine Janis Pohontsch, Carola Bindt, Inga Becker-Hebly

**Affiliations:** 1 Department of Child and Adolescent Psychiatry, Psychotherapy, and Psychosomatics, University Medical Center Hamburg-Eppendorf, Martinistraße 52, W29, Hamburg, 20246 Germany; 2 Department of General Practice and Primary Care, University Medical Center Hamburg-Eppendorf, Martinistraße 52, W37, 20246 Hamburg, Germany

**Keywords:** Contraception, Reproductive health, Healthcare, Counseling, Barriers to care, Transgender, Gender dysphoria, Interview study, Provider perspectives

## Abstract

**Background:**

Gender-affirming hormones do not completely suppress fertility in transgender and gender diverse individuals, highlighting the need for counseling on pregnancy risk and contraceptive options. However, research on current contraceptive care is limited. Studies from the US have identified several barriers to care as well as facilitators, but no studies on this topic have yet been conducted in Europe. This study examined transgender and gender diverse contraceptive care in Germany from the perspective of healthcare providers, assessing its importance and their roles in it, as well as exploring barriers and facilitators.

**Methods:**

Thirty semistructured qualitative interviews with German healthcare providers were conducted between December 2023 and February 2024. The interview guide included questions on contraceptive care for transgender and gender diverse individuals. Data were analyzed using structuring qualitative content analysis (Kuckartz) with deductive and inductive category development.

**Results:**

Most interviewees highlighted the need for contraceptive care, depending on various factors such as the type of gender-affirming care applied, sexual practices, and transgender and gender diverse individuals’ desire for pregnancy prevention. Half of the interviewees also offered contraceptive care (depending on their specialization). Numerous barriers to contraceptive care, such as a lack of awareness of contraceptive needs, insufficient research and training programs for medical staff, have been reported, highlighting the importance of facilitators for care such as the implementation of contraceptive counseling as a standard protocol.

**Conclusion:**

To improve contraceptive care for transgender and gender diverse individuals, the establishment of clear structures and responsibilities, more research, and qualifications among the involved specialties are needed. To gain a comprehensive understanding of the care situation, future research should include the perspectives of transgender and gender diverse individuals.

**Trial registration:**

We obtained the approval of the Hamburg Medical Council (“Ärztekammer Hamburg”) to conduct our study by means of a “scientific case” (2023-300381-WF, September 11th 2023).

**Supplementary Information:**

The online version contains supplementary material available at 10.1186/s12978-025-02223-7.

## Abstract (plain language summary)

Transgender and gender diverse people experience persistent incongruence between their experienced gender identity and their sex assigned at birth - diagnosed as gender incongruence. Many use gender-affirming hormones, such as testosterone or estrogen, to match gender identity and body. These hormones decrease the ability to get pregnant but do not stop it completely. That’s why it is important to give this population information about pregnancy risks and contraceptive options. So far, most research on this topic has come from the US, but little is known about the situation in Europe. This study looked at how healthcare providers in Germany think about contraceptive care for transgender and gender diverse people and how they experience care provision.

Thirty healthcare providers (medical doctors and psychologists) in Germany were interviewed between December 2023 and February 2024. Most healthcare providers said they believed contraceptive care was important for transgender and gender diverse people, depending on factors like the type of hormones used and sexual practices, and half of the healthcare providers also offered contraceptive care. Some common challenges included a lack of knowledge about contraception for transgender and gender diverse people, lack of research, and limited training of medical staff. Healthcare providers said that better contraceptive care for transgender and gender-diverse people in Germany needs more research, clear protocols or guidelines, as well as more training for healthcare providers. Future studies should also ask transgender and gender diverse people themselves about their needs and experiences.

**Supplementary Information:**

The online version contains supplementary material available at 10.1186/s12978-025-02223-7.

## Background

Every person should have the right to bodily autonomy and reproductive freedom, including the right to prevent unintended pregnancies, supported by access to accurate information and resources. However, these rights are often compromised for transgender and gender diverse individuals (TGDI). The present study aims to examine the current state of contraceptive care for TGDI in Germany. TGDI experience persistent incongruence between their experienced gender identity and their sex assigned at birth - diagnosed as gender incongruence [1] or, when accompanied by psychological distress, gender dysphoria [2]. Most TGDI wish to align their physical appearance with their gender identity, which often involves seeking gender-affirming care through hormonal or surgical interventions [3].

Gender-affirming interventions impact fertility, making it essential to support TGDI in accessing fertility preservation and to provide counseling on their options and limitations. Although gender-affirming hormones can significantly reduce fertility, they do not lead to complete infertility, meaning that contraception remains a critical need for TGDI [4]. The risk for unintended pregnancy is reinforced by the generally higher barriers TGDI face in accessing health care services – such as abortion services – compared to cisgender individuals (when gender identity aligns with sex assigned at birth) [4–6]. In addition, the teratogenic effects of testosterone underscore the importance of offering contraceptive care and providing information about the need to discontinue testosterone use if pregnancy occurs [3, 4].

Sexual activity, particularly vaginal intercourse, is a prerequisite for contraceptive needs. It is shaped by diverse biological, psychosocial, and sociopolitical factors. Historically, TGDI have been falsely stereotyped as hyposexual [7]. Although gender-affirming interventions and gender dysphoria can affect sexual desire and function, a Belgian-Dutch study suggests that overall levels of sexual activity among TGDI do not differ significantly from those of cisgender individuals [8]. A US-American study indicates that TGDI report slightly lower rates of vaginal intercourse but a higher number of sexual partners than cisgender individuals [9]. However, to adequately capture the complexity of TGDI's sexuality, future research should apply validated instruments, improved methodologies, and larger samples [7].

The Standards of Care 8 (SOC8), an international clinical guideline published by the World Professional Association for Transgender Health, clearly recommends the use of contraception for TGDI receiving gender-affirming hormones and provides an overview of available contraceptive methods: For TGDI with testes (male sex assigned at birth), options primarily include mechanical barriers (e.g. condoms), permanent sterilization (e.g.vasectomy), and gender-affirming surgery (e.g. orchiectomy, which also results in sterilization) [3]. In contrast, SOC8 states that TGDI with ovaries (female sex assigned at birth) have a broader range of options [3], including hormonal methods (oral, subcutaneous implant, skin patch, intrauterine, or intravaginal) or nonhormonal methods (intrauterine device, barrier methods such as diaphragm, cervical cap, or internal condom), as well as surgical methods (hysterectomy or sterilization) [10, 11].

Contraception use among TGDI has been found to vary from 20% among transgender men (*n *= 18/89), and 36% among transgender women (*n *= 4/11) with no specification for sexual practice [12], to 87% among gender minorities assigned female at birth with specification for vaginal intercourse [9]. Many TGDI name testosterone as their method of contraception [4, 11, 13–16] and have been partly advised to do so by their healthcare providers (HCPs) [11], indicating inadequate implementation of contraceptive care for TGDI. Moreover, compared with cisgender individuals, TGDI often have distinct contraceptive preferences, favoring methods that alleviate gender dysphoria, induce amenorrhea, and avoid estrogen [6]. In their clinical opinion paper, Krempasky et al. (2020) highlighted additional desirable characteristics of contraceptive methods such as the avoidance of pelvic procedures and breast tenderness as well as the ease of discontinuation and concealment [11].

TGDI in need of contraceptive care face numerous barriers. Due to the understudied interaction between contraception and testosterone [4, 13], some TGDI worry about negative interference of those two [13]. Further fears, such as encountering transphobia in clinical settings and being misgendered, may also discourage TGDI from seeking contraceptive care [6, 13]. These challenges are mirrored by the study results of Unger et al. (2015) showing that only 29% of their surveyed OBGYN (obstetrics and gynecology) providers in the US felt comfortable providing care for male transgender patients and that 35% felt comfortable caring for female transgender patients [17].

HCPs-related barriers have been identified over the past decade by research mainly conducted in the US [11, 14, 15, 18]. For instance, one of the few existing studies (Fix et al., 2020) captured the perspectives of US-American HCPs on contraceptive care and abortion services for TGDI [6]. They identified barriers, such as knowledge gaps regarding contraception for TGDI, a lack of TGDI-sensitive language, and insufficient specific training among HCPs. Studies in the closely related field of fertility preservation have also identified barriers to counseling including access and cost issues as well as provider-related challenges concerning knowledge and patient-related challenges such as patient maturity (age and emotional development), and the challenge of making future-oriented decisions [19, 20]. Some of these obstacles may also affect contraceptive counseling. To address these barriers, several strategies have been proposed. HCPs can contribute to high-quality contraceptive care by being aware of questions and misconceptions about contraception [16], adopting a sensitive, non-offensive attitude during counseling (e.g., becoming aware of microaggressions that should be avoided) [15], creating an inclusive clinical environment and language [13, 14] and reflecting on internalized discriminating behaviour [21].

In summary, previous research on contraceptive care provision for TGDI has identified several barriers and suggested strategies for improvement. While current research has focused on the US, it is unclear to what extent findings are applicable to other countries [3]. Research on contraceptive care remains limited [22]. To our knowledge, no research on contraceptive care for TGDI in Europe has been published, yet. To address this gap, we aimed to explore whether the barriers identified also exist in Germany. Specifically, we examined how HCPs – who may themselves contribute to certain barriers – perceive the current state of contraceptive care for TGDI. This study was guided by the following research questions: Do German HCPs see a need for contraceptive care for TGDI? What role do HCPs themselves play in contraceptive care provision? What barriers and improvement strategies do they identify in contraceptive care for TGDI?

## Methods

This interview study explores the status quo of contraceptive care for TGDI from the perspective of HCPs in Germany using semistructured interviews and qualitative content analysis. The present study is part of a twofold research project on reproductive care for TGDI in Germany, with a focus on contraception on the one hand and fertility preservation on the other hand [23].

### Interview guide

We developed the interview guide using Helfferich’s four-step approach – “collecting, reviewing, clustering, and subsuming” questions –, a method designed to balance openness and structure in qualitative research [24]. In the first step, the collection phase, we adopted questions from the questionnaires used in the publications by Fix et al. (2020), Chen et al. (2019), and Tishelman et al. (2019), who had conducted qualitative interviews on reproductive care for TGDI in the US, and translated them into German. In addition, we compiled further questions aimed at addressing our research questions. In the second step of Helfferich’s method, we reviewed all the questions to assess whether they precisely addressed our research aims and subsequently categorized them according to our four research questions and the two subtopics of contraception and fertility preservation. In the final step, we reduced the number of questions by aiming to cover multiple aspects within single items and by removing redundant questions. Afterwards, we consulted experienced researchers in qualitative and healthcare research, as well as a researcher identifying as transgender, to obtain feedback on both methodological and content-related aspects, as well as to incorporate the perspective of a representative from the target research group (TGDI).

The final semistructured interview guide included questions on both contraception and fertility preservation. For each subtopic, the interview questions explored HCPs’ views on the importance of the specific type of care, their role in its provision, and their perceptions of barriers and facilitators. The present paper focuses on the findings regarding contraceptive care, while results concerning fertility preservation will be published separately [23]. Finally, the interview guide was pilot tested (*n* = 2) within the research group and with a HCP experienced in TGDI healthcare to assess its applicability and comprehensibility. The interview conductors CB and MW took field notes, but no audio recording was carried out. Hence, the pretest data were not included in the final analysis. After pretesting, the interview guide was finalized with minor adjustments. See “Additional file 1” for the complete interview guide.

### Recruitment & participant selection

Participants included HCPs who had experience providing out- or inpatient care for TGDI in Germany and held a medical or psychological degree. We included HCPs caring for TGD children and adolescents, as well as those working with adults. We excluded nurses, midwives, and medical students from this study to focus on HCPs with decision-making power in the means of care. German nurses and midwives, who usually are not trained academically, have little decision-making responsibility for screening and triage processes in healthcare facilities. This may differ in other countries. The inclusion and exclusion criteria are presented in Table 1. All volunteer HCPs who met the inclusion criteria were included in the study. We aimed for a broad distribution of participants across different areas of (both somatic and mental health) expertise, ensuring that multiple cases were represented within each specialty. Study invitations were sent via different newsletters and mailing lists of work groups for transgender healthcare. Additionally, in a second recruitment phase, invitations were sent to potential interview partners who had been recommended for participation in previously conducted interviews.

**Table 1:** In- and exclusion criteria for study participation

Inclusion Criteria	Exclusion Criteria
Experience in healthcare provision to TGDI	No experience in healthcare provision to TGDI
Gynecology, reproductive medicine, urology, andrology, endocrinology, surgery, general practice, pediatrics, psychiatry and psychotherapy	Specialties with no association to reproductive healthcare (such as otorhinolaryngology)
Healthcare provider located in Germany	Healthcare provider from other European countries including German-speaking ones such as Austria and Switzerland
Medical or psychological degree	Midwives, nurses and medical students

*TGDI* transgender and gender diverse individuals

### Ethics & consent

We obtained the approval of the Hamburg Medical Council (“Ärztekammer Hamburg”) to conduct our study by means of a “scientific case” (2023–300381-WF, September 11th 2023). The interview participants were given written information about the study and the data protection policy via email one week before the interview date. Participation was voluntary and no compensation was provided. Consent to study participation was obtained verbally and audio-recorded at the beginning of each interview after all open questions had been addressed.

### Researcher characteristics

The research team consisted of six members: the first and second author, CB (medical doctor, doctoral candidate) and MW (medical student, doctoral candidate), conducted the interviews, transcribed and analyzed the data. Both underwent interview training based on Gläser [25]. At the time of the interview conduction and analysis, both identified as white, abled-bodied, cisgender women in their mid-twenties with their primary occupation being medical students. Their relationship with and motivation for this research topic were influenced by internships in this work field (transgender healthcare and gynecology). Data discussion and review were performed by the research group: IBH, LH (both psychologists, postdoctoral degrees) and CaB (medical doctor, senior lecturer). NP (psychologist, postdoctoral degree, senior lecturer, experienced qualitative researcher) supported this project with methodological consulting.

### Data collection

Data collection took place between December 2023 and February 2024. We conducted 30 semistructured interviews in the German language, each lasting 20 to 60 min (*M* = 40). They were conducted online via the platform Webex or in person, predominantly in a clinical setting, and audio recorded. In most cases, the interview conductor and interview partner met for the first time within the scope of this study. In each interview, we asked the participants questions about both contraception and fertility preservation. In addition to the semistructured interview guide, we used a short demographic questionnaire to collect participant characteristics verbally. After each interview, a protocol comprising field notes was created. The data were saved pseudonymously.

### Transcription & data analysis

For the present analysis, we examined all data regarding contraceptive aspects. The platform F4x, an AI-supported tool, generated the interview transcriptions. Both CB and MW reviewed all transcripts with the audio recordings for alignment and utilised “content-semantic transcription rules” [26] for uniformity and comparability. In the next step, weapplied structuring qualitative content analysis [27] using the MAXQDA software. This analysis method condenses data while preserving its core meaning, integrating prior theories, and allowing new insights by combining data-driven and concept-driven approaches. Based on the interview guide, a deductive coding frame was developed that included code definitions, anchor examples and coding rules, one for each subtopic (contraception and fertility preservation). Both CB and MW coded six selected transcripts (15%) independently, then compared and discussed the codes to establish a shared understanding of the coding frame and to refine coding rules. Subsequently, CB and MW each coded half of the transcripts and exchanged the coded transcripts for revision. Next, we derived inductive subcategories individually for each of the subtopics (contraception and fertility preservation). The coding frame expanded accordingly, repeating the process and preparing thematic summaries. The coding frame for the subtopic contraception concerning this paper is provided in “Additional file 2”. Nearing the analysis of the 30th interview we considered thematic saturation to be reached as no more new inductive categories emerged [28]. Throughout the process, we continuously checked intersubjective comprehensibility, and differences were discussed within the research group to achieve consensus in coding. Results, interview guide and quotes were translated into English for publication.

## Results

### Interviewee characteristics

Participant characteristics are presented in Table 2. We interviewed 30 HCPs from a variety of specialties providing somatic (*n* = 20) and mental healthcare (*n* = 10). The interviewees had 5–38 years (*Mdn* = 15,5 years, SD = 9 years) of work experience in their specialty and provided care to TGDI for 2–28 years (*Mdn* = 13,5 years, SD = 7 years). The proportion of TGDI in relation to the total patient population of the interviewees varied from 1% to 100% (*Mdn* = 30%, SD = 29%). Interviewees reported a slight predominance in providing care to transmasculine individuals over transfeminine individuals. In many cases (*n* = 11), no statement was made regarding the provision of care to non-binary individuals. 13 interviewees reported rarely or never providing care to non-binary individuals, while six stated that they do so frequently. 17 of all interviewees provided care to children and adolescents, and 13 exclusively to adults. 21 interviews were conducted online via the platform Webex and 9 interviews were conducted face-to-face.

**Table 2:** Sociodemographic characteristics of interviewed healthcare professionals (*N* = 30)

**Specialty**	
*Somatic health*	20 (66.6%)
Gynecology	9
Urology	5
Internal Medicine	3
Pediatrics (Endocrinology)	3
*Mental health*	10 (33.3%)
Psychiatry/Psychosomatic Medicine	3
Psychology	7
**Work experience**	
*Years within interviewees‘ specialty*	*Mdn* = 15,5 years (SD = 9 years)
Early-career (0–5 years)	1 (3%)
Mid-career (6–15 years)	14 (47%)
Senior-level (> 15 years)	15 (50%)
*Years with TGDI*	*Mdn* = 13,5 years (SD = 7 years)
1–10 years	14 (46.7%)
11–20 years	11 (36.7%)
21–30 years	5 (16.$\:7$%)
**TGDI clientele**	
*in interviewees’ overall clientele* ^*1*^	*Mdn* = 30% (SD = 29%)
TGDI was the minority of clientele ^2^	22 (73.4%)
TGDI approximately 50/50 ^3^	2 (6.6%)
TGDI was the majority of clientele ^4^	6 (20%)
*TGDI clientele’s gender identity*	
Exclusively/mainly transmasculine	12 (40%)
Exclusively/mainly transfeminine	6 (20%)
Equally transmasculine/feminine/all gender	12 (40%)
* TGDI clientele’s age groups*	
Children and adolescents only (4–21 years)	7 (23.3%)
Predominantly adolescents and adults (≥ 14 years)	10 (33.3%)
Adults only (≥ 18 years)	13 (43.3%)

*TGDI * transgender and gender diverse individuals

^1^ Interviewees estimated these numbers

^2^ 1–40%, ^3^ 41–60%, ^4^ 61–100%

The results section is structured according to the research questions mentioned above and the coding frame that emerged from data analysis. The main codes structuring the results section are “Need for Contraceptive Care”, “Contraceptive Care Provision”, “Barriers to Care” and “Improvement Strategies”.

### Need for contraceptive care

Most interviewees considered contraceptive care important for TGDI, highlighting its equal importance for TGDI and cisgender people. However, a small number of interviewees (*n* = 2) regarded contraception as a marginal issue for TGDI and saw no need for care provision due to infertility and sexual practice.

Interviewees identified several factors influencing the need for contraceptive care:

Type of gender-affirming care applied: Some of the interviewees stated that although fertility is restricted when gender-affirming hormones are applied, they should not be considered a contraceptive method. However, for younger TGDI receiving puberty blockers or when genital gender-affirming surgery (e.g. hysterectomy, penectomy, orchiectomy) has been carried out, interviewees generally saw no need for contraceptive counseling in terms of pregnancy prevention.

*“…depending on whether they have had gender-affirming bottom surgery*,* contraception is necessary.”* (8-MW, para. 66, somatic HCP)^1^

Sexual practices: Some of the interviewees perceived TGDI as having low sexual activity due to gender dysphoria, particularly among TGD adolescents, as puberty can reinforce gender dysphoria. They observed that as TGDI grew older and received more gender-affirming care, they tended to become more interested in exploring their sexuality.

*“So*,* I would say that gender dysphoria initially stands in the way of exploring and actively living one’s sexuality. Therefore*,* the desire to alleviate gender dysphoria and the desire for gender-affirming hormones are foremost to be able to explore sexuality later on.”* (2-CB, para. 66, mental HCP).

In addition to gender dysphoria, interviewees reported that gender-affirming hormones could also affect sex drive, with estrogen commonly perceived as decreasing libido and testosterone as increasing it. Furthermore, TGDI were often perceived as being in partner constellations or engaging in sexual practices that may not carry a pregnancy risk. Nevertheless, some interviewees emphasized that in their practice, constellations with conceptional potential did occasionally occur, making it important to discuss contraception in such cases.

TGDI’s desires: Some interviewees underlined the importance of contraception in cases where there is currently no desire to have children. Two interviewees reported that younger individuals in particular desired reliable pregnancy prevention. They assumed that this preference was often driven by a wish to minimize confrontation with their own biology, regarding fertility and menstruation. Many interviewees perceived TGDI as particularly inclined to seek hormonal oral contraception, primarily to suppress menstrual bleeding.

*“The intention of using the pill probably includes not only the desire for contraception*,* but also the desire for period suppression*,* so that the two [goals of contraception] merge.”* (2-CB, para. 46, somatic HCP).

Awareness of the need for contraception: Interviewees described the awareness for the need of contraception among TGDI as heterogeneous. While some were perceived as knowledgeable about contraception, interviewees reported that TGDI may lose sight of the necessity of contraception when starting gender-affirming hormones, and younger TGDI were seen as less rational in their contraceptive use. Consequently, the importance of contraceptive counseling for this group was highlighted.

*“The younger the candidates are*,* the more important counseling actually becomes.”* (3-CB, para. 84, mental HCP).

Prevention of sexual transmitted infection (STI): Although STI prevention was not the main focus of our study, it was repeatedly mentioned as an important reason for contraceptive counseling, particularly in the context of specific sexual practices (e.g. anal sex) or multiple sex partners. Interviewees further stated that pregnancy prevention and STI prevention were interconnected, as certain contraceptive methods, such as condoms, served both purposes.

*„I recommend to simply use a condom. Then you kill two birds with one stone*,* so to speak. It provides protection for sexually transmitted diseases and conception.”* (17-MW, para. 56, somatic HCP).

Contraceptive method: One interviewee saw little need for contraceptive counseling, arguing that some contraceptive methods could be used without medical assistance. In contrast, two others highlighted the potential benefit in contraceptive counseling, especially for oral hormonal contraceptives, due to the complexity of different hormonal constellations (regarding gender-affirming care) and individual physical reactions (insufficient suppression of menstrual bleeding).

### Contraceptive care provision

When investigating care provision, we distinguished between contraceptive counseling and the prescription of contraceptive methods. Interviewees reported that their contraceptive care provision focused primarily on TGDI with ovaries, although some also offered contraceptive care to individuals with testes.

Half of the interviewees reported that they actively addressed contraceptive counseling in their healthcare provision for TGDI.

*“We always provide information about contraception*,* primarily about the need for contraception*,* before the start of the treatment [application of gender-affirming hormones].”* (5-CB, para. 50, somatic HCP).

Their contraceptive counseling included the following topics:

Assessment of the contraceptive need: Some interviewees reported that they assessed the contraceptive care needs by discussing reproductive intentions, sexual activity, anatomic characteristics and the desire for menstrual suppression.

Provision of contraceptive education: Key issues in HCPs’ contraceptive education concerned the implications of gender-affirming care for contraceptive needs: namely, that testosterone does not serve as a contraceptive, that the absence of menstrual bleeding or sperm ejaculation does not indicate complete infertility, and that testosterone may increase libido.

Discussion of contraceptive methods: Interviewees mentioned that in their contraceptive counseling, they primarily recommended the use of oral hormonal contraception – particularly the progestin pill for menstrual suppression – and barrier methods, primarily condoms. While most considered oral hormonal contraception a useful option, one interviewee considered this method counterproductive, and another considered it appropriate only for TGDI not receiving gender-affirming hormones. Intrauterine device implantation was frequently mentioned. Less commonly cited methods included morning-after pill, hysterectomy, vasectomy, menstrual cycle tracking, and GnRH injections. 

Half of the interviewees stated that they did not provide contraceptive counseling. However, two of them conceded that they would perform contraceptive counseling if TGDI raised the issue.

*“I have to say quite frankly that this is an issue that would be discussed if the patient or their accompanying partner brings it up.”* (9-CB, para. 72, somatic HCP).

Only a few HCPs were actively involved in prescribing contraceptive methods to TGDI, depending on their specialization. However, there remained variation in the extent and nature of care offered. One interviewee attributed their abscence of contraceptive care provision to their primary work with adolescents. Two interviewees pointed out that contraceptive care was not part of their professional remit.

This divergence in practice raises questions about who should bear responsibility for contraceptive care. When asked, more than half of the interviewees (*n* = 17) identified gynecology as the primary responsible specialty. However, one interviewee noted that many TGDI face difficulties in seeking gynecological care, making it essential for other specialties to help bridge this gap.

*“We don’t see many people who are willing to go there [gynecology]. So*,* it’s our job to do that [provide contraceptive care]. And accordingly*,* every medical intervention or hormone therapy that we carry out should include this [contraceptive care].”* (12-CB, para. 51, somatic HCP).

Endocrinology was regarded as the second most important specialty in providing contraceptive care, particularly in terms of educating about the need for contraception and the fact that testosterone should not be used as a contraceptive. General practitioners were also mentioned as important and practical contraceptive care providers because they see TGDI regularly and could provide guidance on changing contraceptive needs. Psychiatry and psychological care were seen as responsible for addressing sexuality and, in this context, including the discussion on the need for contraception. Urology was considered to play a minor role. Three interviewees suggested that each party involved in TGDI healthcare should participate in contraceptive care provision to an extent that is suitable for their specialty. However, there was some ambiguity regarding care responsibility: some interviewees explicitly pointed out that endocrinology (*n* = 1), urology (*n* = 2), psychiatry (*n* = 5) and general practice (*n* = 2) should not be responsible for contraceptive care provision.

### Barriers to care

Interviewees identified barriers to care for TGDI both at an individual and at a structural level.

#### Individual barriers

TGDI-related barriers perceived by the interviewees included discomfort in discussing sexuality – and consequently contraception – with their HCPs, a lack of awareness for the contraceptive need and avoidance of gynecological appointments. According to the interviewees, TGDI often wished to avoid confrontation with their own bodies, particularly with dysphoria-inducing body parts, which often include the genitals.

*“A gynecological appointment*,* the visibility of one’s own genitals - this is something that triggers many*,* many fears and leads to the circumstance that most people under the age of 18 have never seen a gynecologist.”* (14-MW, para. 62, mental HCP).

Interviewees reported that HCPs likewise felt uncomfortable discussing sexuality with TGDI and often lacked awareness of the contraceptive need. Reasons given for this lack included the belief that gender-affirming hormones provided sufficient contraception, the assumption that TGDI were not sexually active, the historical practice of forced sterilization of TGDI (as a requirement for legal gender recognition in Germany until 2011 which resulted in irreversible infertility) and the fact that some HCPs had not considered the issue prior to the interview.

*“One barrier is perhaps my ignorance – that I haven’t really put this [contraceptive care] on my agenda yet.”* (13-CB, para. 68, somatic HCP).

Additionally, a certain reservation and insecurity towards TGDI among HCPs were mentioned, as were a lack of knowledge and understanding of the diversity of gender identities and sexual orientations.

*“And there’s also the lack of education*,* that they [gynecologists] think*,* ‘Huh*,* you’re a man now*,* you don’t want to have vaginal intercourse anymore.’ or ‘Why do you still have your reproductive organs if you want to be a man now?’. Or another common one: ‘Why are you living in a homosexual relationship with a cis man when this all would be easier as a cis woman?’.”* (11-CB, para. 108, somatic HCP).

The absence of an open, nonjudgmental attitude towards TGDI was perceived as contributing to negative experiences with HCPs, such as deadnaming, misgendering, and discrimination.

#### Structural barriers

In addition to individual barriers, interviewees identified numerous structural barriers, most of which concern general healthcare for TGDI and consequently hinder contraceptive care. Interviewees claimed that there was little literature available to guide high-quality healthcare with clear guidelines, textbooks and interdisciplinary networks missing. Furthermore, they highlighted a lack in training programmes for gender-sensitive healthcare, resulting in a shortage of well-trained, gender-sensitive medical staff.

*“I think*,* there are not enough offers for low-threshold training courses for practitioners to learn this [gender-sensitive care].”* (6-MW, para. 50, somatic HCP).

Interviewees noted that gynecological care – and healthcare in general – was not gender-sensitively configured. Interviewees saw a shortage in care offerings for TGDI, particularly those being TGDI-sensitive and close to TGDI’s residential environment. Additionally, a lack of transparency was held responsible for difficulties in finding TGD-specific care offerings.

*“For trans individuals*,* there is sometimes far too little clarity. ‘Who is responsible for me? Where can I get empathetic and high-quality counseling and treatment?’”* (6-MW, para. 96, somatic HCP).

Another limitation to high quality care mentioned by interviewees was the insufficient time available for TGDI-specific counseling. The cost for contraceptive methods for TGDI was also seen as a barrier to accessing contraception, as expenses are not covered by health insurance in Germany.

### Improvement strategies

Interviewees suggested that education on gender diversity and an increase in its visibility could reduce the stigmatization of TGDI and create social acceptance. This might encourage TGDI to seek contraceptive care and support HCPs to develop an open and friendly attitude towards TGDI. Enhanced education could be achieved through greater social media presence of HCPs sharing content of their work with TGDI, as well as educational initiatives and public health campaigns.

*“…visibility of life realities of trans people in cultural products*,* media*,* school*,* children’s books*,* yes*,* early normalization*,* accepting narratives*,* so to speak.”* (8-CB, para. 107, mental HCP).

Furthermore, interviewees considered the integration of TGDI healthcare into medical education alongside continuous training for trainees and specialists, to be essential. Additionally, according to the interviewees, an improvement in the ease of discussing contraception and sexuality of HCPs was needed.

*“…definitely regular training in the curricula. And not just for special interest initiatives*,* but knowledge about minorities and the realities of life for marginalised groups in general.”* (8-CB, para. 107, mental HCP).

Resources such as counseling centers, textbooks, and publications in journals of different specialties could aid in improving professional training. Interviewees suggested establishing advisory services and professional associations to support HCPs in TGDI healthcare. More conferences, case reports, best practices examples, and interdisciplinary workgroups are needed for knowledge exchange, addressing research gaps through professional collaboration.

Furthermore, it was suggested that each specialty contributed to contraceptive care from its unique perspective building their care upon each other. Therefore, close cooperation and direct communication between specialties involved in contraceptive care for TGDI are essential. Referrals should be made in cases where TGDI counseling exceeds a HCP’s expertise.

Interviewees stressed the importance of raising awareness of contraceptive needs and providing comprehensive information. They further advocated for the integration of contraceptive care into standard protocol of TGDI healthcare.

*“But actually*,* we should ask this [contraception] in the same standardised and structured way as we do [with other topics].”* (16-MW, para. 70, somatic HCP).

Suggestions included incorporating contraceptive needs into medical history forms and addressing them on an ongoing basis. Interviewees also highlighted the necessity of dispelling misconceptions about contraception for TGDI. Care should follow evidence-based guidelines and be inclusive and diverse. Informative materials, such as leaflets, should be co-developed by TGDI and interdisciplinary medical associations. Interviewees emphasized the need for further research from the perspective of TGDI regarding their wishes and needs for contraceptive care and their experiences within the current healthcare system. Research findings should be easily accessible and published across multiple specialties.

Further improvement strategies included facilitating access to care through better information and greater transparency of available care offerings.

*“It should also be advertised more actively what options are available and how easy it can be.”* (12-CB, para. 63, somatic HCP).

They suggested publishing specific TGDI care services on HCPs’ websites, creating a regional map of trans-sensitive HCPs. Increased transparency would help TGDI navigate specialized care and assist HCPs in making referrals to trained professionals. The creation of peer groups and group therapy were also recommended to enhance knowledge exchange among TGDI.

The organization of care affects accessibility. Interviewees advocated for integrated care centers combining all TGDI-related specialties and decentralized services to ensure local availability. For better information management and to reduce repetitive patient questioning, one interviewee proposed the establishment of a national patient registry. Interviewees called for shorter waiting time for trans-sensitive care and the establishment of open-access counseling. More time and billing options for contraceptive counseling were also needed according to the interviewees.

## Discussion

### Main findings

This study aimed to contribute to the scientific discussion on reproductive justice by providing first insight in the contraceptive care situation for TGDI in Germany. We assessed HCPs’ views on the importance of contraceptive care for TGDI, their role in providing it, and their perceptions of barriers and facilitators to contraceptive care. Most HCPs recognized the need for individualized contraceptive care for TGDI, and half of them also provided contraceptive counseling, addressing both contraceptive needs and options. There was no consensus among the interviewees on which specialties beyond gynecology should be involved in contraceptive care provision. Reported barriers included a lack of awareness, a discomfort discussing sexuality, and a lack of research and training. The suggested improvement strategies highlighted the need to enhance contraceptive care in Germany through increased awareness, standardized care protocols, and training for HCPs.

### Strength & limitations

This study provides a first explorative insight into HCPs’ perceptions of contraceptive care across various specialties with significant experience in providing healthcare for TGDI in Germany. However, this study also has limitations. Its qualitative focus on HCPs’ perspectives offers a one-sided and nonrepresentative view of contraceptive care in Germany. The historic exclusion of TGD communities from knowledge production has left critical information absent from scientific literature, fostering ignorance and reliance on cisnormative assumptions [29]. Stereotypical gender norms and implicit assessments of gender conformity can shape HCPs’ evaluations [30]. We must assume that such factors have influenced our findings. However, our research did not aim to identify or analyze provider biases influencing access to care, and these biases were therefore not explicitly revealed in the results. Incorporating TGDI's perspectives into future research as well as an intersectional analysis addressing overlapping oppressions—including cisnormativity, sexism, transmisogyny, classism, racism, marginalization of sexual minorities, and ableism— [31] is crucial to address knowledge gaps, challenge systemic biases, reduce stigma, and improve the quality and accessibility of healthcare. Furthermore, future quantitative studies are needed to assess the scope and impact of the issues identified in this study. Participation bias likely influenced our results, as HCPs with greater interest or experience in TGDI healthcare were presumably more inclined to take part. This self-selection may have skewed the findings toward a more favorable portrayal of current care practices, underrepresenting the perspectives of less experienced providers who may offer less adequate contraceptive care. To mitigate these limitations, we aimed to maximize diversity by ensuring participants were distributed heterogeneously in terms of years in practice and specialties. We did not account for gender identity, race, or geographical work setting (urban or rural) in our sociodemographic characteristics, which limits our ability to analyze potential differences across these demographics. The design of the interview guide also poses a limitation due to the ordering of questions, with fertility preservation addressed before contraception. As a result, in some interviews, less time was available to explore issues regarding contraception in depth. Additionally, when discussing contraception, interviewees tended to refer to their responses concerning fertility preservation rather than considering contraception-specific aspects. A further limitation in the study design concerns the lack of a clear definition regarding TGDI healthcare experience as criterion for interview participation. As a result, participants reported a wide range of experiences, both in terms of years working with TGDI and the proportion of TGDI relative to cisgender clientele. These reported percentages offer limited insight into the actual number of TGDI seen. Therefore, we recommend that future research includes a standardized definition of TGDI healthcare experience and collects absolute numbers of TGDI seen per defined time frame (e.g., per month) to improve comparability. To ensure transparency regarding researcher bias, we have provided details on the research team and potential influencing factors, such as gender, research experience, and motivation. All members of the research team understand themselves as allies to TGDI and advocate for gender-affirming care and reproductive rights of all genders.

### Discussion of results and comparison with existing literature

Our findings suggest that contraceptive needs among TGDI are shaped by gender-affirming care, sexual practices, reproductive intentions, and STI prevention, varying with age, gender and anatomy. These patterns align with prior research emphasizing tailored counseling across gender identities, sexual orientations, and transition stages [4, 32]. Interviewees reported three contraceptive intentions—alleviating gender dysphoria, preventing pregnancy, and suppressing menstruation—aligning with those identified by Fix et al. (2020). Krempansky et al. (2020) described four additional contraceptive characteristics valued by TGDI, complementing our findings.

In the present sample, contraceptive counseling for TGDI appeared to focus primarily on assessing and educating about contraceptive needs and available options. A few interviewees reported actively prescribing contraceptives. While participants represented various medical specialties, contraceptive care was not always part of their professional remit. However, no differentiation was made in the analysis, further restricting the applicability of results to the wider HCP population. In addition, iterviewees also expressed differing views regarding care responsibilities: whether specialties beyond gynecology should provide contraceptive care and whether responsibility lies solely with somatic or also mental HCPs. Opinions also varied on the benifit of oral hormonal contraception for TGDI. HCPs could use the Standards of Care 8 as a clinical guidance for contraceptive care as they provide clear recommendations regarding the use of contraception, different contraceptive options, and how to address the need for menstrual suppression [3]. However, the Standards of Care 8 highlights the research gap concerning the interaction between gender-affirming hormones and contraception as well as their safety profile. Since it is an international guideline intended to be adapted to local contexts, responsibilities for care may need to be defined individually in accordance with each country’s healthcare system and clinical guidelines. The gap between care-seeking behavior – TGDI avoiding gynecological appointments – and responsibility for care provision – gynecology as primary provider – highlights the need to discuss gender-inclusive practices within gynecology. Additionally, trained specialists, including mental health professionals, could bridge this gap, though proper training to competently address sexual healthcare needs is essential. Overall, clearly defining care responsibilities and fostering interdisciplinary collaboration is essential to closing this gap in care. Further investigation of TGDI’s perspectives is necessary to assess whether their needs are being met by the current provided care.

Individual barriers identified by our interviewees, namely the limited awareness regarding contraceptive needs, partially align with previous research. Misconceptions, such as viewing testosterone as a sufficient contraceptive [11] and the perception of TGDI as hyposexual – rooted in historical narratives [7] – were echoed in our findings. Uniquely, some HCPs linked the limited awareness in Germany to the country’s history of compulsory sterilization for legal gender recognition (until 2011). The recognition of reproductive rights in Germany may still be influenced by this history, potentially leading some HCPs to more or less consciously assume that TGDI are – or even should be – infertile and incapable of having children. As a result, contraception may not be offered. This could also have a lasting impact in other countries with similar histories. The individual and structural barriers identified in this study for Germany align with those found in an US-American study, including a lack of awareness for the contraceptive need, insufficient care offerings, inadequate training of HCPs, and binary, cis- and heteronormative healthcare environment [6]. Especially, internalized cisnormative assumptions and transphobia among HCPs may lead to discriminatory practices and act as barriers to accessing healthcare [21, 30].

Improvement strategies suggested by our interviewees addressed these barriers: raising awareness, integrating contraceptive care as standard protocol, establishing training on TGDI healthcare for medical professionals, and improving the availability and transparency of care offers. These suggestions align with previous literature emphasizing the elimination of microaggressions [15], the establishment of an inclusive clinical environment [6, 13, 14] and education on gender identity [6]. An interview study with HCPs on discrimination experiences in trans-specific healthcare suggests mandatory seminars for HCPs on self-awareness regarding their own gender identity [21]. The mentioned barriers and facilitators may also affect closely related reproductive and sexual healthcare issues, such as abortion services, fertility and pregnancy care, and STI prevention. Further research is required to ascertain their impact.

### Clinical implications

HCPs may gain support for future contraceptive counseling by taking into account factors influencing the contraceptive care need (age, gender, type of gender-affirming care applied, sexual practices and the desire to have children or prevent pregnancy) and individuals’ contraceptive intentions. Evidence-based guidelines would benefit from incorporating these factors and offering sexual healthcare counseling tailored to individual needs—particularly with regard to hormonal profiles. Meeting the desires for contraception among TGDI is essential for high-quality care provision. Furthermore, the establishment of gender-inclusivity in healthcare – particularly in gynecological care – is needed as well as a better education and training for medical staff (including reflecting on internalized discriminating attitudes and behaviour). Interestingly, some interviewees reported discomfort in discussing sexuality in both TGDI and HCPs. This aspect highlights the necessity of not only improved training for professionals, but also broader societal acceptance of discussion about sexuality and reinforcement of general sexual health education – particularly regarding gender diversity and sexual orientations. Furthermore, the structural barriers and improvement strategies identified in our study suggest the need to expand care offerings, particularly in residential proximity for TGDI and integrated care centers. Research integrating TGDI’ perspectives in addition to those of HCPs is crucial to explore contraceptive preferences, side effects, and their alignment with TGDI healthcare needs. As STI prevention was closely linked to pregnancy prevention in our study sharing common influencing factors, future research on contraception for TGDI should integrate STI prevention considerations. Barriers to pregnancy prevention may similarly affect STI prevention efforts, as well as other closely related fields of reproductive and sexual healthcare, such as abortion services, fertility and pregnancy care. Further research is needed to assess their impact, including perspectives from midwifery and nursing staff, which may provide additional valuable insights into care.

## Conclusion

Most HCPs recognized a need for contraceptive care for TGDI in Germany. However, they also identified numerous barriers and suggested strategies for improvement. Advancing gender inclusivity in contraceptive services, expanding care options, and enhancing professional training and inclusive sexual education are crucial to addressing existing barriers and ensuring equitable access to care. Key recommendations include establishing clear structures, defining responsibilities, and promoting interdisciplinary collaboration among providers. The development of specific guidelines that incorporate these recommendations is essential to improving contraceptive care for TGDI in Germany, just as future research that integrates TGDI perspectives and addresses their specific contraceptive needs.

## Supplementary Information

Additional file 1: Interview Guide Trans*Fertil. This document provides an insight into the interview guide used for this study.

Additional file 2: Coding Frame from Structuring Qualitative Content Analysis (Kuckartz). This file provides an overview of the coding frame used to analyze the 30 interview transcripts using Kuckartz’s (2018) method of “structuring qualitative content analysis”.

Additional file 3*: *COREQ Checklist. This file provides an overview of the quality criteria from the COREQ Checklist by [33] used to assess methodological quality, including references indicating where the corresponding information can be found in the paper.

## Data Availability

The datasets generated and analyzed during the current study are not publicly available due to reasons of data protection and the need to protect the identities of our interview participants, but they are available from the corresponding author on reasonable request.

## Abbreviations

TGDI: Transgender and gender diverse individuals
HCPs: Healthcare providers
STI: Sexual transmitted infection
OBGYN: Obstetrics and gynecology

## Footnotes

1 Reference information for each quotation includes: the interview label (interview number + interviewer abbreviation), the paragraph number of the quotation, and the type of care provided by the interviewee.
