# Supplementary material for: Contraceptive care for transgender and gender diverse individuals from the perspective of healthcare providers in Germany: a qualitative study
Source: Reprod Health. 2025 Dec 6;22:250. doi: 10.1186/s12978-025-02223-7 (PMC12706887; doi:10.1186/s12978-025-02223-7)
Supplement: Supplementary file 1 — Additional file 1: Interview Guide Trans*Fertil. This document provides an insight into the interview guide used for this study. [file 12978_2025_2223_MOESM1_ESM.docx]

# **Additional File 1**

# **Interview Guide Trans*Fertil**

# *Disclaimer: In our survey, we would like to include both transgender and gender diverse individuals. However, we will use the term “trans individuals” for the sake of linguistic flow, meaning trans* as an umbrella term including gender diverse individuals.*

# ***Demographic Questions***

- What is your highest level of education?
- What specialty do you work in? Do you have a sub-specialisation?
- What kind of care do you offer: out- or in-patient care? Somatic or mental healthcare?
- How long have you been working in your specialty?
- What proportion of your patients are trans individuals? Please estimate the percentage!
- How many trans individuals do you see per week?
- Since when do you provide healthcare to trans individuals?
- Which trans population do you primarily care for: Transmasculine, transfeminine or non-binary? Children, adolescents or adults?
- What age are the trans individuals you care for? Please give an age range!
- With what concerns do trans individuals come to you?
- What is the approximate proportion of trans individuals who express a need for reproductive care? Please estimate the percentage!

# ***Open Interview Questions***

1. What comes to your mind when you think of “reproductive care for trans individuals”?

# *Importance of Reproductive Care: Fertility Preservation*

1. How important do you think fertility care is for trans individuals?
   1. Why do you consider fertility care for trans individuals as relevant/less relevant?

# *Provision of Reproductive Care: Fertility Preservation*

1. Do you provide fertility care to trans individuals yourself? If yes: What is your contribution to this care?
   1. Counseling: Can you tell a bit more about your counseling?
      1. Timing of counseling?
      2. What topics do you address?
         1. Desire to have children
         2. Methods of fertility preservation
         3. Risks of fertility preservation
      3. How would you describe the decision-making process? Who is involved?
      4. Do you use information material in your counseling?
      5. Which specialty should provide counseling for fertility preservation for trans individuals?
   2. Do you perform fertility preservation methods?
      1. If yes, which?
      2. If no, do you refer to experts of fertility and reproductive medicine?
   3. Do you offer fertility treatments to induce a pregnancy in trans individuals? If yes, which?
2. How would you describe the current fertility care situation for trans individuals and its accessibility in Germany?
3. Please estimate: How many trans individuals proceed with fertility preservation after counseling?
   1. Why do you think trans individuals do not seek fertility preservation?
   2. Why do you think fertility preservation counseling is sometimes not offered?
4. Please estimate: How many trans individuals proceed with fertility treatment to induce a pregnancy?
   1. Why do you think trans individuals do not seek these treatments?
   2. Why do you think fertility treatment counseling is sometimes not offered?

# *Importance of Reproductive Care: Contraception*

1. How important do you think contraceptive care is for trans individuals?
   1. Why do you consider contraceptive care for trans individuals as relevant/less relevant?

# *Provision of Reproductive Care: Contraception*

1. Do you provide contraceptive care to trans individuals yourself? If yes: What is your contribution to this care?
   1. Counseling: Can you tell a bit more about your counseling?
      1. Timing of counseling
      2. What topics do you address?
      3. How would you describe the decision-making process? Who is involved?
      4. Do you use information material in your counseling?
      5. Which specialty should provide contraceptive counseling for trans individuals?
   2. Do you prescribe or provide contraceptive methods? If yes, which?
2. How would you describe the current contraceptive care situation for trans individuals and its accessibility in Germany?
3. Please estimate: How many trans individuals use contraception after counseling?
   1. Why do you think trans individuals do not use contraception?
   2. Why do you think contraceptive counseling is sometimes not offered?

# *Evaluation of Research and Knowledge*

1. How do you rate the current state of reproductive health research regarding trans individuals?
   1. What topics are well investigated? Please mention examples!
   2. What research gaps do you perceive?
2. How well informed are you about trans individuals’ reproductive health? What topic would you like to know more about?

# *Barriers to Reproductive Care*

1. Do you perceive barriers to fertility care for trans individuals?
   1. If yes, which?
      1. Self-related barriers
      2. Trans individual-related barriers
      3. Access-related barriers
      4. Structural barriers
2. Do you perceive barriers to contraceptive care for trans individuals?
   1. If yes, which?
      1. Self-related barriers
      2. Trans individual-related barriers
      3. Access-related barriers
      4. Structural barriers

# *Improvement Strategies*

1. Do you have ideas on how to improve fertility and contraceptive care?
2. What would optimal interdisciplinary care for trans individuals look like in terms of fertility preservation and contraception?
   1. What care offers would be part of an optimal fertility care for trans individuals?
   2. What care offers would be part of an optimal contraceptive care for trans individuals?

# *Final Questions*

1. Is there anything else you would like to add regarding the topics of this interview that we haven’t covered yet?
2. Do you have any new thoughts or insights as a result of this interview you would like to share?
3. Are there any other questions we should include in this interview guide?

*After ending the audio recording:*

1. Are there any colleagues or healthcare providers of trans individuals that we should invite to participate in our interview study?

*Note.* We did not ask all sub questions in each interview. We always asked the main questions, while the sub questions served as inspiration to explore the interviewees insight in case they gave a short answer.
For interview development we adopted questions from the questionnaires used in the publications by Fix et al. (2020), Chen et al. (2019), and Tishelman et al. (2019), who had conducted qualitative interviews on reproductive care for TGDI in the US, and translated them into German.
