# Supplementary material for: Contraceptive care for transgender and gender diverse individuals from the perspective of healthcare providers in Germany: a qualitative study
Source: Reprod Health. 2025 Dec 6;22:250. doi: 10.1186/s12978-025-02223-7 (PMC12706887; doi:10.1186/s12978-025-02223-7)
Supplement: Supplementary file 2 — Additional file 2: Coding Frame from Structuring Qualitative Content Analysis (Kuckartz). This file provides an overview of the coding frame used to analyze the 30 interview transcripts using Kuckartz’s (2018) method of “structuring qualitative content analysis”. [file 12978_2025_2223_MOESM2_ESM.docx]

**Additional File 2**

*Coding Frame from Structuring Qualitative Content Analysis (Kuckartz)*

| **Code** | **Content Description** | **Implementation Guideline** | **Key Example (Quotation)** |
| --- | --- | --- | --- |
| **Need for care** | Evaluation of the need for care and its relevance for TGDI | Is coded, when interviewee gives a statement on the need for care and reasons for this evaluation, coded on statements pro and against the need for care | "It's exactly the opposite with trans boys. It is the case that gender-affirming care with testosterone leads to an increase in libido, to increased sexual fantasies and sexual contacts, also shown in research, so that it is always an issue." (2-CB, para. 4, somatic health) |
| ***Evaluation of the importance*** | Evaluation of the importance for contraceptive care for TGDI | Is coded, when interviewee gives a statement about their evaluation of the importance for contraceptive care for TGDI | “Exactly the same as for any cis person, so very relevant.” (11-CB, para. 98, somatic health) |
| ***Factors influencing care needs*** | Factors that influence the need for contraceptive care for TGDI | Is coded, when the interviewee points out factors influencing the need for care. Those can be reenforcing and also attenuative factors for the need for care | “…that testosterone is not a contraceptive and therefore additional ovulation inhibition must be applied.” (12-CB, para. 49, somatic health) |
| *Gender affirming care* | Impact of gender affirming care on the need for contraceptive care | Is coded when interviewee explains how and which gender affirming care influences the need for contraceptive care | “This combination of antiandrogen and oestrogen is not a recognised contraceptive option.” (9-CB, para. 68, somatic health) |
| *Sexual activity* | Influence of the sexual activity on the need for care and its reasons | Is coded when interviewee explains how and to what extend sexual activity and sexual practices influence the need for care depending on choice of partner, libido, age differences, gender dysphoria | “Under puberty blockers and subsequent gender-affirming care with oestrogen, sexual fantasies and desire as well as sexual activity decrease.” (12-CB, para. 48, somatic health) |
| *TGDI‘s awareness for contraception* | TGDI awareness for the necessity of contraceptive application | Is coded when interviewee gives a statement on the awareness of TGDI for contraception, positive or negative | “For some patients it is crystal clear that contraception is absolutely necessary despite gender-affirming hormone therapy.” (5-CB, para. 50, somatic health) |
| *Aim of contraception use* | Reasons for TGDI to use contraception | Is coded when interviewee reports about their experiences why TGDI use contraception: no desire to have children, abortions, menstrual suppression | “So, yes, it is important to use specific contraception if there is no desire to have children.” (7-CB, para. 54, somatic health) |
| *Miscellaneous: STI* | STI prevention as an additional reason for contraceptive care | Is coded when the interviewee reports on the importance of STI prevention as a reason for contraceptive care | “Depending the sexual practice, it is highly relevant for people who have changing sexual partners.” (10-CB, 63, somatic) |
| **Implementation of care** | Contribution to contraceptive care by the interviewee | Is coded when interviewee reports about their own work and contribution to contraceptive care: decision-making process, information material, as well as specific contraceptive measures and counseling | “I would say that we educate about the fact that testosterone is no conception prevention.” (12-CB, para. 28, somatic health) |
| ***Content of care*** | Concrete content and offers of the contraceptive care | Is coded when interviewee talks about content, meaning concrete topics and questions addressed in contraceptive counseling and procedures and drugs that are prescribed | “We always provide information about contraception, primarily about the need for contraception, before the start of the [application of gender-affirming hormones] treatment.” (5-CB, para. 50, somatic health) |
| ***Structure of care*** | Organisation and structure of contraceptive care | Is coded when interviewee talks about how contraceptive care is organized: at what point contraceptive care is offered, how often contraceptive counseling is implemented, reasons why contraceptive care might not be implemented | „I think I have to say quite frankly that this is an issue that I think would more likely to discuss with if the patient or the partner were to bring it up.” (9-CB, para. 72, somatic health) |
| ***Responsibility for care*** | Perspectives on responsibility of contraceptive care depending on the speciality and task | Is coded when interviewee speaks about which specialty provides or should provide which part of contraceptive care | “I think the gynecology. But I see it rather in the outpatient sector, as for normal population.” (7-CB, para. 58, somatic health) |
| ***Care seeking behavior*** | TGDI's usage behavior and demand for contraceptive care | Is coded when the interviewee talks about to what extend TGDI seek out and demand contraceptive care and what questions they rise regarding contraceptive methods | “So, I can't imagine and wouldn't understand if someone that received contraceptive counseling refuses contraceptive measures. In this respect, I estimate the proportion [of contraceptive usage among TGDI] to be high, 90 percent.” (7-CB, para. 62, somatic health) |
| **Barriers** | Reasons for hinderence of implentation and usage of care | Is coded when interviewee talks about reasons why contraceptive care cannot be implemented or why and what kind of difficulties may rise | “It is difficult for trans men to go to a gynecological practice. They almost unanimously have negative experiences, in terms of the language used, deadnaming, and the whole experience.” (12-CB, para. 51, somatic health) |
| ***Individual*** | Barriers that arise through human behavior | Is coded when interviewee talks about barriers that are caused by human behavior | “The problem on the side of the healthcare provider: for whatever reason, provider don't have the courage to bring it up or think it would be out of place or perhaps have reservations about bringing it up.” (9-CB, para. 96, somatic health) |
| *TGDI related* | Barriers that are brought up by TGDI in the usage of contraceptive care | Is coded when interviewee mentions barriers which are brought up by TGDI | “The only thing is that you have to make your way to the gynecologist somehow. And I think that's perhaps a hurdle, because it's also problematic for many trans men to have check-ups at all and to present themselves as men to the gynecologist.” (7-CB, para. 60, somatic health) |
| *Healthcare provider related* | Barriers that are brought up by the healthcare providers in the implementation of care | Is coded when interviewee mentions barriers which are brought up by healthcare provider | “One barrier is perhaps my ignorance, that I haven't really put this on my agenda yet.” (13-CB, para. 68, somatic health) |
| ***Structural*** | Barriers that are brought up through organisational structures and the healthcare system | Is coded when interviewee talks about barriers caused by the organisation of the healthcare system | “Many just don't know where to find it[care].” (10-CB, para. 81, somatic health) |
| **Improvement strategies** | Ideas leading to an improvement of contraceptive care | Is coded when interviewee describes ideas for improving contraceptive care for TGDI | “There should also be access to care in residential proximity. So decentralised, very decentralised care.” (8-CB, para. 109, mental health) |
| ***Individual*** | Strategies that lead to an improvement of care through a change of human behavior | Is coded when interviewee talks about improvement strategies that rely on a change of behavior of people (that can be healthcare providers, TGDI, society) | “In any case, regular training in the curricula [of medical students]. And not just for special interest initiatives, but knowledge about minorities and the realities of life of marginalised groups in general.” (8-CB, para. 107, mental health) |
| ***Fascilitating access*** | Strategies that lead to an improvement for care through facilitated access to care | Is coded when interviewee talks about ideas to improve the access for TGDI to contraceptive care | “I think it would be important to create nation-wide structures in which providers who offer care or have expertise can connect, more networking. So that everyone who works in psychosocial work also knows where they [healthcare provider] can refer them [TGDI] to” (2-CB, para. 96, mental health) |
| ***Content*** | Strategies that lead to an improvement of care through change or an extension of the care content | Is coded when interviewee talks about ideas how to shape care services in the terms of content | “Basically, it would be important for all practitioners to be aware that fertility protection and contraceptive counseling must be part of the standard healthcare for trans people.” (8-MW, para. 103, somatic health) |

*Note.* This table provides an overview of the coding frame used to analyze the 30 interview transcripts using Kuckartz’s (2018) method of “structuring qualitative content analysis”, supported by the MAXQDA software.
Reference information for each quotation includes: the interview label (interview number + interviewer abbreviation), the paragraph number of the quotation, and the type of care provided by the interviewee.
TGDI = transgender and gender diverse individuals
